# INTERDISCIPLINARY COLLABORATION TO SUPPORT AGE FRIENDLINESS AND PREPARATION FOR INTERGENERATIONAL PROGRAMMING

**DOI:** 10.1093/geroni/igae098.3421

**Published:** 2024-12-31

**Authors:** Kelly Munly, Jared Frederick, Harriett Gaston

**Affiliations:** Penn State Altoona, Altoona, Pennsylvania, United States; Penn State Altoona, Altoona, Pennsylvania, United States; Penn State Altoona, Altoona, Pennsylvania, United States

## Abstract

Human Development and Family Studies (HDFS) and History Programs at a small campus collaborated to support Adult Development and Aging students (N=72) to understand the life course context of older adults they prepared to work with in a long-term care practicum experience. This effort to prepare students for intergenerational programming is part of a larger effort toward Age Friendliness at this campus and in the surrounding community. Life course theory is the foundation of project aims and design. After HDFS faculty established a plan for collaboration with the History Program, they were able to provide two classes of Adult Development and Aging students (N=72) with historical insights on the life course experiences of the older adults that students would be working with. Context and life course experiences discussed included dynamics related to diversity—often considered invisible—resulting from the Great Migration, as well as socioeconomic moments in time that would also influence the life course for multiple generations. After each class of discussion facilitated by the History Program, students were asked to provide reflections on their Canvas course online platform on what they had heard in light of their imminent practicum experiences. Initial thematic analysis of student reflections suggest that discussions facilitated between the History and Human Development and Family Studies Programs across two classes enhanced student understanding and supported a stronger foundation toward Age Friendliness and readiness to engage in intergenerational programming.

